# The Bidirectional Relationship between Positive Mental Health and Social Rhythm in College Students:A Three-Year Longitudinal Study

**DOI:** 10.3389/fpsyg.2017.01119

**Published:** 2017-06-30

**Authors:** Dan Cai, Meixia Zhu, Muyu Lin, Xiao Chi Zhang, Jürgen Margraf

**Affiliations:** ^1^Department of Psychology, Shanghai Normal UniversityShanghai, China; ^2^Department of Clinical Psychology and Psychotherapy, Mental Health Research and Treatment Center, Ruhr-Universität BochumBochum, Germany

**Keywords:** social rhythm, positive mental health, cross-lagged panel analysis, college students, longitudinal study

## Abstract

Social rhythm refers to the general regularity of engaging in basic social activities during the week, and was found to be associated with individuals’ positive mental health. The present study investigated the relationship between social rhythm and emotional well-being in a cohort of 2,031 college students over 3 years with a cross-lagged longitudinal panel design. Results revealed that regularity of social rhythm positively predicted emotional well-being in the following year, and vice versa, when the level of both factors in the previous year was controlled. Our study provides evidence of a longitudinal positive reciprocal relationship between social rhythm and positive mental health in younger adult populations.

## Introduction

It is well-known that physical health and psychological health are closely linked with each other. For instance, regular biological daily patterns, such as temperature fluctuations and the circadian rhythm play a key role in maintaining good mental health, whereas disruption or irregularity in time-cues that trigger one’s biological and social behavior, such as changes in the duration of daylight, sleep/wake cycles, social contacts, working schedules, and environment signals, can lead to increasing symptoms of mental illnesses (Ehlers et al., 1988; Costa, 1996; Thase et al., 2002; Grandin et al., 2006; Gorwood, 2012; Lieverse et al., 2013).

Among all physical and social factors related to mental health, the influence of social rhythm patterns – rhythmic social and behavioral patterns in daily routines that may be directly or indirectly related to other people such as mealtimes, bedtimes, and social interaction during weekdays and weekends – might be underestimated, but in fact they have repeatedly been shown to be related to individuals’ mental health (Ehlers et al., 1988; Sylvia et al., 2009). Previous studies regarding social rhythm mainly focused on its relationship with psychopathology, especially with depression (Brown et al., 1996; Lieverse et al., 2013) and bipolar disorder (Grandin et al., 2006; Meyer and Maier, 2006; Bullock et al., 2011). For instance, individuals with bipolar spectrum disorders experienced greater disruption of their social rhythm than controls following both positive and negative life events (e.g., the birth of an infant or losing a spouse or job) (Boland et al., 2012). Further, less social rhythm regularity was found to predict more rapid reoccurrence of affective episodes (i.e., less time in a euthymic state) in bipolar individuals during the prospective follow-up (Shen et al., 2008). And, as proposed in the Social Zeitgeber Theory (Ehlers et al., 1988), disruption of one’s time cues that prompt human circadian rhythms changes the stability of social and biological rhythms, resulting in more symptoms or episodes of mental illness. On the other hand, promoting life rhythm in therapy was also proven effective in treating bipolar disorder, marked by fast stabilization, longer episode-free periods, and decreased disorder recurrence (Frank et al., 2005; Nusslock and Frank, 2012). Beyond affective disorders, there are indications of a relationship between social rhythm irregularity and posttraumatic stress disorder (PTSD). Similarly, many patients with PTSD experience disturbances in their sleep pattern (Harvey et al., 2003; Kobayashi et al., 2007; Haynes et al., 2016), and psychotherapy aiming to enhance social rhythm regularity has demonstrated promising results in reducing symptoms and increasing sleep quality in these patients (Haynes et al., 2016).

As reviewed above, a solid body of research has shed light on the understanding of the bidirectional relationship between social rhythm and mental illness, especially affective diseases. According to the World Health Organization (World Health Organization, 2004), however, good mental health is not only the absence of psychopathology, but additionally “*a state of well-being in which the individual realizes his or her own abilities, can cope with the normal stresses of life, can work productively and fruitfully, and is able to make a contribution to his or her community*” (p.12). In the recent decades, the positive aspects of mental health are taking a more prominent role in public mental health care and disorder-related research and practice. Accumulating studies have shown that the negative and positive aspects of mental health are only moderately related to each other, and that the association between the two is bidirectional, supported by the two-dimensional model of mental health (Westerhof and Keyes, 2010; Kendler et al., 2011; Weich et al., 2011; Lamers et al., 2015). Furthermore, positive mental health emerged as a predictor of the remitting course of mental disorders (Lukat et al., 2017) and the remission of suicidal thoughts in young women after 17 months (Teismann et al., 2016).

To our knowledge, only a few studies have investigated the relationship between social rhythm and positive mental health. For instance, a cross-sectional study with a representative German sample of over 7,000 people revealed that social rhythm irregularity was associated with less life satisfaction and greater depression, anxiety, and stress (Velten et al., 2014). A recent large-scale research study with samples from Russia, Germany, and the United States demonstrated that social rhythm regularity was an important cross-cultural factor in predicting higher positive mental and physical health, as well as less health problems (Margraf et al., 2016). Similarly, a relatively regular working schedule is positively linked to psycho-social well-being, such as family and social commitments. Moreover, individuals who engaged in regular physical activity displayed not only decreased mood symptoms but also enhanced experience of well-being (Penedo and Dahn, 2005). Sleep hygiene, which involved regular sleep schedules, was found to partially or indirectly predict depression and poor well-being in college students (Peach et al., 2016).

Most previous studies focusing on social rhythm and positive mental health adopted a cross-sectional design (Velten et al., 2014; Margraf et al., 2016), leaving the question unresolved whether the strong association found between these two constructs was due to the fact that a regular social rhythm would lead to a higher level of positive mental health, or vice versa. Logically, the next step should be to investigate the longitudinal and potential causal relationship between the two important constructs (Kraemer et al., 1997). The few longitudinal studies so far mainly focused on either the predictor role social rhythm plays on negative mental health (Frank et al., 2005; Shen et al., 2008; Nusslock and Frank, 2012), or, in the opposite direction, how mental illness symptoms predict social rhythm (Boland et al., 2012), suggesting a bidirectional relationship between social rhythm and psychopathological symptoms. Meanwhile, it remains unknown whether social rhythm regularity and positive mental health would also benefit from each other over time.

Therefore, the present study aimed to gain insight into the reciprocal relationship of social rhythm regularity predicting higher levels of positive mental health, and vice versa. The Brief Social Rhythm Scale (BSRS) (Margraf et al., 2016) and Positive Mental Health Scale (PMHS, Lukat et al., 2016) were applied in a large college student sample in China over three, sequential 12-month periods. The BSRS is a new tool with multiple language versions that quickly assesses rhythmicity in eating, sleeping, and socializing patterns. The PMHS was applied, as a valid short unidimensional measure of general emotional well-being (Lukat et al., 2016). A large-scale population diary study revealed that the highest rhythmicity was found within older people and those who cohabite with a partner and children, while younger single populations were relatively less rhythmic (van Tienoven et al., 2014). Given that the academic pressures and life changes of college life may also lead to greater risk of irregularity in social rhythm as well as to changes in emotional well-being, a college student sample was selected for the current study. Furthermore, the survey continued for 3 years in order to track changes in both social rhythm and positive mental health over time.

Based on earlier empirical evidence that social rhythm and positive mental health were associated with each other cross-sectionally (Margraf et al., 2016) and that social rhythm shared a possible reciprocal relationship with mental illness (Frank et al., 2005; Shen et al., 2008; Boland et al., 2012; Nusslock and Frank, 2012), we hypothesized that (1) baseline (T1) social rhythm regularity would positively correlate with baseline positive mental health score; (2) scores on BSRS at T1 would predict scores on BSRS at 1-year-follow-up (T2), and T2 BSRS scores would predict 2-year-follow-up (T3) BSRS scores; (3) PMHS at T1 would also predict PMHS at T2, and PMHS at T2 would predict PMHS at T3; (4) baseline social rhythm would predict change in PMHS at T2, as well as baseline PMHS would predict social rhythm at T2; and (5) change in social rhythm at T2 would predict change in PMHS at T3, and vice versa that PMHS at T2 would predict social rhythm at T3.

## Materials and Methods

### Participants

This project was a part of Bochum Optimism and Mental Health (BOOM) research project (Velten et al., 2014; Margraf et al., 2016). All participants were students at Shanghai Normal University, China. This university offers 17 major programs, the current project randomly selected students from five of the programs, including majors from humanities, sciences, etc. The project lasted for three academic years (from 2013 to 2015) with one distribution of questionnaires each year (altogether three waves of surveys, referred as T1, T2, and T3, respectively). Each survey was conducted in May, which was around the middle of the second semester of the academic year, in order to avoid potential influences of the beginning or end of a new academic year or new semester on the psychological or social well-being of participants. The sample size for T1 was 2,985; 2,992 for T2, and 2,818 for T3. Participants who completed less than 80% of the two target scales, who were suspected not to respond sincerely (i.e., all the answers were the same; e.g., chose 1 in all 9 items of PMHS), or who missed one or more surveys were excluded. Finally, answers from 2,031 participants (68.04% of the initial sample) were included for further statistical analyses. Missing values were replaced by mean values. Among them, 1,621 were females, and 410 were males. At baseline, 1,177 students were in their freshman year, 851 were sophomore year students, and 3 were in their junior year of college. The average age (at T1) of the longitudinal sample was 19.89 ± 0.91, ranging from 17 to 29.

### Measures

#### The Brief Social Rhythm Scale (BSRS)

The BSRS assesses the regularity of participants’ 10 basic daily activities, such as mealtimes, bedtimes, and walking times during the work week/school week and on the weekend (Margraf et al., 2016). General regularity of each activity is rated with a 6-point scale ranging from 1 (*very regularly*) to 6 (*very irregularly*). Items of the BSRS can be found in Appendix A. Final scores are recoded reversely, therefore higher scores of BSRS indicate higher regularity in social rhythm. The internal consistency was 0.80, 0.86, and 0.89, for T1, T2, and T3 based on the responds of the longitudinal sample, respectively.

#### Positive Mental Health Scale (PMHS)

The PMHS is a 9-item scale measuring a holistic concept of emotional well-being related to positive mental health (Lukat et al., 2016). Participants respond on a 4-point Likert-type scale, ranging from 0 (*I disagree*) to 3 (*I agree*). Items of the PMHS can be found in Appendix B. This scale has been applied in several different samples (students and different patient samples), showing strong psychometric properties and a one-dimensional structure. In a major validation study on student, patients, and general samples, PMHS showed a 1-week retest reliability of 0.81 (general adults sample) and an internal consistency of 0.93 (student sample) (Lukat et al., 2016). The Cronbach’s α of the longitudinal sample was 0.88 (T1), 0.90 (T2), and 0.94 (T3).

### Data Collection

The study was approved by the Ethics Committee of the Faculty of Psychology of the Ruhr-Universität Bochum and the Academic Ethics Committee of the Shanghai Normal University. Study aims, anonymity, voluntariness, and other information of the survey were informed in the advertisement of the study spread across the college campus. Students who agreed with the terms would voluntarily come to the computer rooms of the university within a certain time range specified in the advertisement to participate the study. When they entered the computer rooms, the aims of the study, voluntariness, and anonymity information were explained again by survey conductors, who were two trained graduate students majoring in psychology. Participants gave oral consent one by one, those who did not give consent left the computer room directly. Then, the survey conductors opened the webpage of the online questionnaire for participants to fill in. The online questionnaire did not ask for information related to participants personal ID. Therefore, no other written consent forms were exchanged. This consent procedure was approved by the Academic Ethics Committee of the Shanghai Normal University.

Participants completed the survey in different waves (50–80 per wave) during the same day. Demographic information questionnaire, PMHS, BSRS, and other questionnaires from the BOOM project were used for each survey. Completing the entire questionnaire battery took approximately 30 min. Participants gave their consent before participation and received a gift (about $ 1.5) after completing each survey.

### Statistical Analysis

Correlations between social rhythm and positive mental health (at T1, T2, and T3) were computed as a primary test for the relationship between the two target variables. Then, the mean changes in social rhythm and positive mental health across the 3 years were tested via repeated-measures multivariate analysis of variance (MANOVA) using SPSS (version 19.0; IBM Corp, 2010).

Scores on BSRS and PMHS at T1, T2, and T3 were included for modeling and examining intra-individual changes over time using a cross-lagged panel analysis. The panel analysis was performed using Amos22.0 (Arbuckle, 2013). Cross-lagged path coefficients (i.e., predictive associations) between T1 and T2 for social rhythm (measured by BSRS) and positive mental health (indexed by PMHS) were included as well as the path coefficients between T2 and T3. The panel model further included stability coefficients between T1, T2, and T3 for SBRS and PMHS in order to allow follow-up measurements (T2 and T3) to reflect residual change over time. We further included correlations (non-direction al associations) between social rhythm and positive mental health at each of the three time points (for T2 and T3, correlations were between residual errors) and the correlation between social rhythm at T1 and at T3 as well as the correlation between positive mental health at T1 and at T3 (Model 1). Overall model fit was assessed using chi square test, comparative fit index (CFI), and standardized root mean square residual (SRMR). CFI ≥ 0.95 and SRMR ≤ 0.08 together indicate good model fit (Hu and Bentler, 1999). Alpha level of significance of all path coefficients and correlations was set at 0.05.

In order to examine the potential influence of gender, another two multi-group cross-lagged panel analyses were conducted and compared. In Model 2, the structures of both gender groups were identical as in Model 1, and all the path coefficients were freely estimated. Then in Model 3, the structures were identical with Model 2, but all the respective path coefficients were constrained to be equal across both gender groups. χ^2^ differences were computed to determine whether Model 2 and Model 3 differed from each other.

## Results

### Relations between Positive Mental Health and Social Rhythm

The *means* and *standard deviations* (*SDs*) of the BSRS and the PMHS scores at the three time points are described in **Table 1**. Bivariate correlations among the scores of PMHS and BSRS in 3 years are shown in **Table 1**, indicating that scores from the PMHS and BSRS were significantly correlated per time point (i.e., at T1, *r* = 0.33) and across time points (e.g., for T1 PMHS and T2 BSRS, *r* = 0.22).

**Table 1:** *Means* and *standard deviations* (*SDs*) descriptions and correlations between positive mental health and social rhythm at each time point.

	*Mean*	*SD*	PMHS (T1)	PMHS (T2)	PMHS (T3)	BSRS (T1)	BSRS (T2)
PMHS (T1)	21.91	4.19					
PMHS (T2)	20.53	4.38	0.52^∗∗^				
PMHS (T3)	21.07	4.64	0.43^∗∗^	0.48^∗∗^			
BSRS (T1)	46.87	6.78	0.33^∗∗^	0.25^∗∗^	0.20^∗∗^		
BSRS (T2)	46.32	7.57	0.24^∗∗^	0.33^∗∗^	0.20^∗∗^	0.46^∗∗^	
BSRS (T3)	45.24	7.95	0.20^∗∗^	0.23^∗∗^	0.36^∗∗^	0.37^∗∗^	0.44^∗∗^


PMHS, Positive Mental Health Scale; BSRS, Brief Social Rhythm Scale; T1, baseline; T2, 1-year-follow-up; T3, 2-year-follow-up; ^∗∗^*p* < 0.01.

### Changes in Positive Mental Health and Social Rhythm across Time

A repeated-measures MANOVA was conducted with the time (T1, T2, and T3) as within group independent variable and the scores on the BSRS and scores on the PMHS as dependent variables. The Mauchly’s test of sphericity was violated, therefore Huynh-Feldt epsilon correction was applied. Bonferroni correction was used for pairwise comparisons.

For PMHS, a significant effect of time was observed, *F*(1.97,3991.03) = 96.29, *p* < 0.0001, η_p_^2^= 0.05. Pairwise comparisons showed that PMHS scores at T1 was higher than the scores at T2, *Mean*_difference_ = 1.38, *SE*_difference_ = 0.09, *p* < 0.0001, *d* = 0.32, and at T3, *Mean*_difference_ = 0.84, *SE*_difference_ = 0.11, *p* < 0.0001, *d* = 0.19; and that the scores of PMHS at T2 was lower than that at T3, *Mean*_difference_ = -0.55, *SE*_difference_ = 0.10, *p* < 0.00001, *d* = 0.12.

For BSRS, a significant effect of time was found, *F*(1.97,3998.08) = 43.05, *p* < 0.0001, η_p_^2^ = 0.02. Further analyses indicated that the social rhythm regularity decreased significantly year by year: the social rhythm regularity at T1 was more regular than that at T2 (*Mean*_difference_ = 0.55, *SE*_difference_ = 0.17, *p* = 0.003, *d* = 0.07) and at T3 (*Mean*_difference_ = 1.62, *SE*_difference_ = 0.18, *p* < 0.0001, *d* = 0.22). In addition, the social rhythm measured at T2 was also more regular than measured at T3, *Mean*_difference_ = 1.08, *SE*_difference_ = 0.18, *p* < 0.0001, *d* = 0.14.

### Cross-Lagged Panel Modeling

**FIGURE 1:**
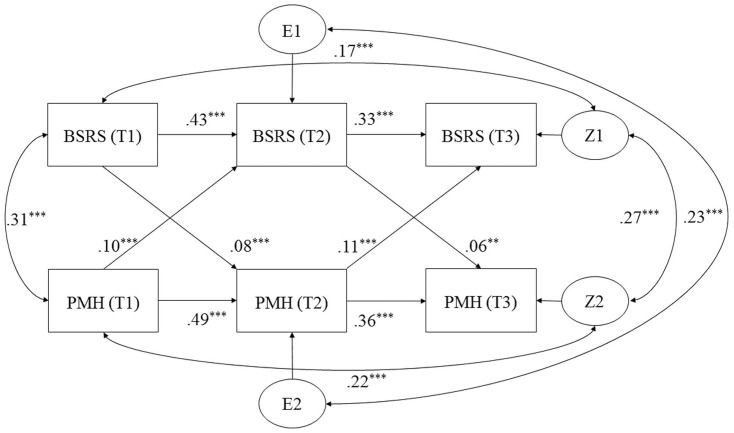
Cross-lagged panel model for social rhythm measured by Brief Social Rhythm Scale (BSRS) and positive mental health measured by Positive Mental Health Scale (PMHS). T1, baseline; T2, 1-year-follow-up; T3, 2-year-follow-up; double-headed arrow, correlation; one-head arrow, regression. E1, E2, Z1, and Z2 represent the residual errors for BSRS (T2), PMHS (T2), BSRS (T3), and PMHS (T3), respectively. ^∗∗^*p* < 0.01; ^∗∗∗^*p* < 0.001.

The PMHS and BSRS were simultaneously examined in a parallel process using cross-lagged panel Model 1 (**Figure 1**). The model was a good fit to the data, χ^2^ (2) = 34.01, *p* < 0.0001, CFI = 0.99, and SRMR = 0.029. The standardized path coefficients of the model are provided in **Figure 1**. As expected for BSRS, the autoregressive regression estimates were positive, strong, and significant from T1 to T2 and from T2 to T3. The same was found for PMHS from T1 to T2 and from T2 to T3, indexing also by positive, strong, and significant autoregressive regression estimates. The cross-lagged parameter estimates of social rhythm at T1 and T2 to PMHS at T2 and T3, respectively, were positive and significant at 0.01 and 0.001 level, suggesting that social rhythm regularity could predict positive mental health even when controlling for the scores from the previous years. Meanwhile, the path coefficients from PMHS at T1 and T2 to BSRS at T2 and T3, respectively, were also positive and reached a 0.001 level of significance, indicating that positive mental health was also a strong predictor of social rhythm in college students.

### Potential Gender Effect

After dividing the sample into female and male subgroups, Model 2 and Model 3 were conducted based on both gender groups. Model fit comparison results showed that both models did not significantly differ from each other, Δχ^2^ = 8.42, *df* = 8, *p* = 0.39. This result indicated that the path coefficient values in both gender groups were similar, thus the results of Model 1 were representative for both genders.

## Discussion

The present study was the first study to examine the bidirectional relationship between social rhythm and positive mental health over 3 years using cross-lagged panel analysis in a college student sample. The large sample size (*N* = 2031) in our study provided high power to detect even small effects. Results showed that social rhythm regularity decreased year by year, while emotional well-being seemed to fluctuate from decreasing at T2 and then increasing again at T3. As expected, our analyses further revealed a significant reciprocal relationship between social rhythm and positive mental health, indicating that social rhythm regularity predicted the level of positive mental health a year later, and vice versa. These findings broaden the cross-sectional results in earlier studies and extend the understanding of the relationship of social rhythm and emotional well-being in younger adults.

The present study is innovative in several ways. First, as expected, social rhythm regularity was a strong predictor of positive mental health, suggesting that improving social rhythm may help to increase psychological well-being in college students. One explanation could be that enhanced rhythm regularity would directly promote one’s mental well-being. For instance, several studies have pointed out that a regular daily rhythm would benefit sleep (Ohayon et al., 2001; Morgan, 2003), and in turn good quality sleep may increase the positive aspects of mental health, such as subjective well-being (Hamilton et al., 2007; Peach et al., 2016). An alternative but not incompatible explanation is that social rhythm regularity would decrease the level of mental symptoms or chances of onset of mental illness (Ehlers et al., 1988; Grandin et al., 2006; Shen et al., 2008; Bullock et al., 2011; Nusslock and Frank, 2012; Lieverse et al., 2013), promoting better mental health (Lamers et al., 2015). For example, it has been shown that social factors such as shift work and irregular working hours may desynchronize normal circadian rhythms (Costa, 1996), elevate stress response (Ulhoa et al., 2011), and lead to mental problems such as chronic fatigue, anxiety (Cole et al., 1990), and depressed mood (Driesen et al., 2010). Thus, a relatively regular schedule may lower the negative aspect of body and mental health, and lead to better positive mental health. Future research is warranted to clarify the potential mediators (e.g., changes in biological rhythm, increase in good sleep quality, or decrease in mental illness) of this bidirectional relationship.

Secondly, the reciprocal relationship displayed that positive mental health was also a consistent predictor of social rhythmicity. This result was consistent with some earlier findings, such as that positive affect and eudaimonic well-being are directly associated with better sleep quality and may buffer the impact of negative psychosocial factors (Steptoe et al., 2008), and that aging women with higher levels of eudaimonic well-being had longer duration of REM sleep compared to those showing lower levels of eudaimonic well-being (Ryff et al., 2004).

Furthermore, while the majority of previous studies used positive mental health only as an outcome measure indexing a desired state of mind (Steptoe et al., 2008), having positive mental health and emotions also proved to have many benefits, such as enhancing job performance in the following year (Cropanzano and Wright, 1999), protecting individuals against physical decline in old age (Ostir et al., 2000), and predicting longevity (Diener and Chan, 2011). However, only a few studies have considered positive mental health as predictive factor (Teismann et al., 2016; Lukat et al., 2017). Our empirical results add to this by pointing out that higher level of positive mental health helps to increase social rhythm regularity which was found to be associated with better mental state and less mental illness (Margraf et al., 2016).

In addition, the present study provided a picture of how psychological well-being and social rhythm fluctuate in college students across time. On the one hand, the results indicated that this population is indeed at risk of increasing irregularity in their daily rhythm as they proceed with their studies, which is in line with previous studies showing that younger and single populations were more irregular in their social rhythm compared to older people or to those living with a partner and children (van Tienoven et al., 2014). On the other hand, the current findings indicated that the psychological well-being in college students fluctuated over the years, and on a similar level in German, Russian, and United States samples reported in an earlier study (Margraf et al., 2016). These results also suggested that mental health of college students is not a fixed factor but can change over time (Xin et al., 2012), emphasizing the importance of additional longitudinal surveys regarding this topic.

There are some limitations to the current study. First, the sample included only college students, therefore, the results cannot be generalized to other adult populations without caution. Second, positive mental health consists of more than just emotional well-being (Baumeister et al., 2013), and “well-being” itself is composed of different aspects, such as social well-being or life-satisfaction. Consequently, when various components of positive mental health are considered, a differential pattern of the relationship between social rhythm and positive mental health might unfold (Lamers et al., 2012; Bieda et al., 2016). Future studies should address the various components of positive of mental health and well-being. Third, a large majority of the longitudinal sample was female, which could impact the representativeness of the results. However, the model comparisons demonstrated that the gender factor did not appear to have a significant impact on the final results.

## Conclusion

In sum, the current study provides preliminary evidence of a mutually enhancing relationship between social rhythm and positive mental health with a 3-year longitudinal design in a large college student sample. Further studies should take other components of positive mental health into account and consider a more comprehensive picture of this reciprocal relationship and its stability.

## Author Contributions

All authors agree to be accountable for the content of the work. DC designed the study and organized data collecting. MZ collected data, did the main statistical analyses, and draft part of the manuscript. ML did part of the statistical analyses and wrote and revised the manuscript. XZ organized data collecting and supervised statistical analyses. JM designed the study and the project, and reviewed the paper.

## Conflict of Interest Statement

The authors declare that the research was conducted in the absence of any commercial or financial relationships that could be construed as a potential conflict of interest.
