# Supplementary material for: The Bidirectional Relationship between Positive Mental Health and Social Rhythm in College Students:A Three-Year Longitudinal Study
Source: Front Psychol. 2017 Jun 30;8:1119. doi: 10.3389/fpsyg.2017.01119 (PMC5492866; doi:10.3389/fpsyg.2017.01119)
Supplement: Supplementary file 1 [file Data_Sheet_1.docx]

## Appendix A. Brief Social Rhythm Scale.

|  |  | 非常规律(very regularly) | 相对规律(quite regularly) | 有些规律(somewhat regularly) | 有些不规律(somewhat irregularly) | 相对不规律(quite irregularly) | 非常不规律(very irregularly) |
| --- | --- | --- | --- | --- | --- | --- | --- |
| 1 | 周一到周五睡觉时间  (Going to bed Monday to Friday) | 1 | 2 | 3 | 4 | 5 | 6 |
| 2 | 周末睡觉时间  (Going to bed on weekends) | 1 | 2 | 3 | 4 | 5 | 6 |
| 3 | 周一到周五起床时间  (Getting up Monday to Friday) | 1 | 2 | 3 | 4 | 5 | 6 |
| 4 | 周末起床时间  (Getting up on weekends) | 1 | 2 | 3 | 4 | 5 | 6 |
| 5 | 周一到周五与学习或工作中的人会面  (Meeting other people at the university or at work Monday to Friday) | 1 | 2 | 3 | 4 | 5 | 6 |
| 6 | 周末与学习或工作中的人会面  (Meeting other people at the university or at work on weekends) | 1 | 2 | 3 | 4 | 5 | 6 |
| 7 | 周一到周五在闲暇时间与其它人会面  (Meeting other people during time off Monday to Friday) | 1 | 2 | 3 | 4 | 5 | 6 |
| 8 | 周末在闲暇时间与其它人会面  (Meeting other people during time off on weekends) | 1 | 2 | 3 | 4 | 5 | 6 |
| 9 | 周一到周五饮食  (Having regular meals Monday to Friday) | 1 | 2 | 3 | 4 | 5 | 6 |
| 10 | 周末饮食  (Having regular meals on weekends) | 1 | 2 | 3 | 4 | 5 | 6 |

## Appendix B. Positive Mental Health Scale.

|  |  | 不符合(do not agree) | 稍微不符合(tend to disagree) | 稍微符合(tend to agree) | 完全符合(agree) |
| --- | --- | --- | --- | --- | --- |
| 1 | 我经常都有轻松和愉快的心情。  (I am often carefree and in good spirits.) | 🞏1 | 🞏2 | 🞏3 | 🞏4 |
| 2 | 我享受我的生活。  (I enjoy my life.) | 🞏1 | 🞏2 | 🞏3 | 🞏4 |
| 3 | 总而言之，我满意我的生活。  (All in all, I am satisfied with my life.) | 🞏1 | 🞏2 | 🞏3 | 🞏4 |
| 4 | 一般情况下，我是自信的。  (In general, I am confident.) | 🞏1 | 🞏2 | 🞏3 | 🞏4 |
| 5 | 我能很好地满足自己的需求。  (I manage well to fulfill my needs.) | 🞏1 | 🞏2 | 🞏3 | 🞏4 |
| 6 | 我处在良好的身体和精神状态中。  (I am in good physical and emotional condition.) | 🞏1 | 🞏2 | 🞏3 | 🞏4 |
| 7 | 我感觉我能处理好生活和生活中出现的困难。  (I feel that I am actually well equipped to deal with life and its difficulties.) | 🞏1 | 🞏2 | 🞏3 | 🞏4 |
| 8 | 我做的事情中，很多事情能给我带来快乐。  (Much of what I do brings me joy.) | 🞏1 | 🞏2 | 🞏3 | 🞏4 |
| 9 | 我是一个性格沉稳、平衡的人。  (I am a calm, balanced human being.) | 🞏1 | 🞏2 | 🞏3 | 🞏4 |
